# The Effect of Corporate Social Responsibility and Public Attention on Innovation Performance: Evidence from High-polluting Industries

**DOI:** 10.3390/ijerph16203939

**Published:** 2019-10-16

**Authors:** Wei Wang, Xue-Zhou Zhao, Feng-Wen Chen, Chia-Huei Wu, Sangbing Tsai, Jiangtao Wang

**Affiliations:** 1 School of Management and Engineering, Nanjing University, Nanjing 210093, China; wwqd2hs@smail.nju.edu.cn (W.W.); xzhao@nju.edu.cn (X.-Z.Z.); 2 School of Economics and Business Administration, Chongqing University, Chongqing 400030, China; 3 Institute of Service Industries and Management, Minghsin University of Science Technology, Hsinchu 300, Taiwan; 4 University of Electronic Science and Technology of China Zhongshan Institute, Zhongshan 528400, China; sangbing@zsc.edu.cn (S.T.); jiangtao-w@foxmail.com (J.W.)

**Keywords:** corporate social responsibility, public attention, innovation performance, environmental benefit, environmental quality, high-polluting industries, technology

## Abstract

High-polluting industries are important sources of pollutant emissions, and closely related to many environmental issues. High-polluting firms face the pressure to exploit technological innovation for improving their environmental operations. This paper explores the impact of corporate social responsibility and public attention on the innovation performance of high-polluting firms. Based on a sample of China’s listed firms in high-polluting industries from 2011 to 2016, we use a panel data model to investigate the associations among corporate social responsibility, public attention and innovation performance. The results show that there is a positive association between corporate social responsibility and innovation performance. There is a positive association between public attention and innovation performance as well. The pressure of regional economies can hinder innovation performance. Furthermore, in the subsample of state-owned enterprises, the association between public attention and innovation performance is more pronounced. Meanwhile, the corporate social responsibility of non-state-owned enterprises plays a stronger role for innovation performance, but its effect will be limited by the pressure of regional economies. Our results can help high-polluting firms implement the innovation strategies for obtaining more environmental benefits and achieving sustainable development.

## 1. Introduction

Environmental issues have attracted serious attention of governments around the world. These problems are closely related to the consumption of natural resources and the pollutant emissions of different industries. Among these environmental issues, high-polluting industries are important sources of pollutant emissions, and can even trigger some extreme weather events, such as sandstorms and smog. A recent study indicates that the frequency of smog in regions with concentrated high-polluting industries is much higher than that in other regions [1]. However, high-polluting firms are also the main driving force for economic growth, especially in developing countries [2]. There is an argument about whether we should protect the environment with restricting the operations of high-polluting firms, or destroy the environment with enhancing the role of high-polluting firms in economic growth. In order to solve this problem, high-polluting firms need to adopt technological innovations for reducing pollutant emissions, such as improving resource utilization and reducing energy consumption [3].

**Figure 1:**
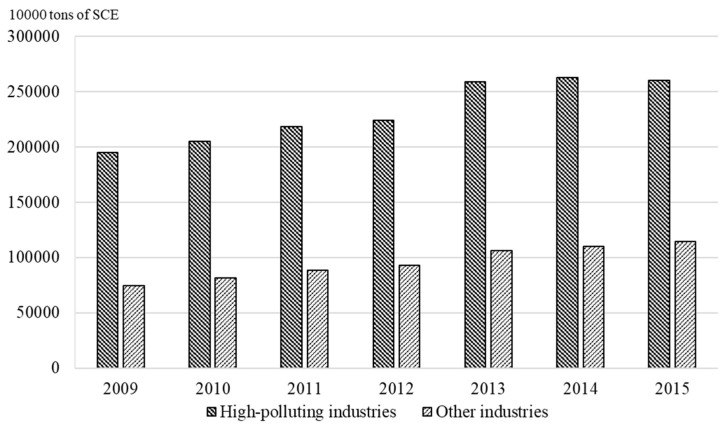
Energy consumption of different industries in China.

**Figure 2:**
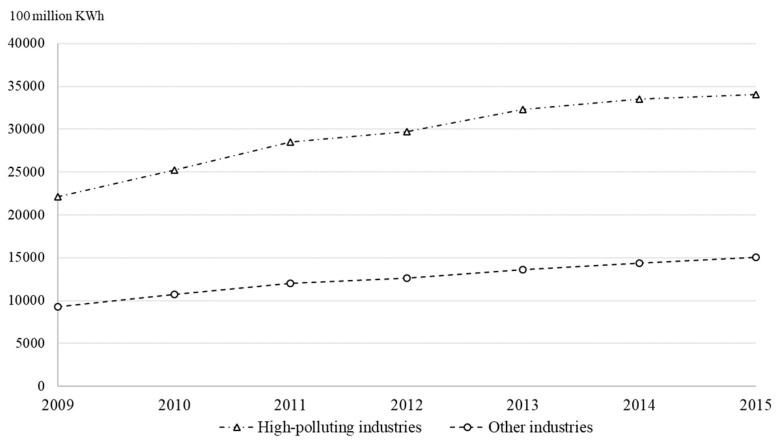
Electricity consumption of different industries in China.

During the economic development of developing countries, high-polluting industries play an important role in economic activities. Among developing countries, China is the biggest economy and has the largest population. High-polluting industries in China include thermal power, steel, metallurgy, building materials, mining and so on, and the contribution of high-polluting industries to China’s gross domestic product (GDP) is 36.1% in 2018 [4]. The local governments demand high-polluting industries to support the development of regional economies, but high-polluting industries can also threaten the ecological environment by pollution emissions [5]. As shown in Figure 1, the energy consumption of China’s high-polluting industries has been rising in recent years [4]. According to Figure 2, the operations of high-polluting industries need to be supported by more and more electricity consumption, and the energy demand of high-polluting industries is much higher than that of other industries [4]. Compared with other developing countries, China pays more attention to sustainable development, and has enacted many laws and regulations for environmental protection, such as the Law of Energy Conservation (2007), the Law of Cleaner Production Promotion (2012), the Law of Air Pollution Control (2018) [6]. Faced with China’s sustainable development strategy, high-polluting firms bear huge pressure from environmental protection. In addition, the proportion of state-owned enterprises in China’s high-polluting industries is relatively large, and they suffer more due to strict environmental policy and energy resource constraints [7]. State-owned enterprises in China can be backed by government, and focus on promoting the image of the central government. However, non-state-owned enterprises rely on the support of investors, and their behaviors in environmental protection can influence investors’ support [8]. In this situation, how to resolve the conflict between environmental protection and environmental damage has become an important issue for China’s high-polluting firms.

In order to realize sustainable development, technological innovation can improve the growth of high-polluting firms with environmental benefits [9]. The outcomes of technological innovation can reduce the pollutant emission and energy consumption of high-polluting firms. In addition, innovation performance can reflect the investments and efforts of firms’ technological innovation in different industries [10]. In this paper, we focus on the outcomes of innovation, and use the number of patent to measure innovation performance. High-polluting firms can benefit from their innovation performance with resource utilization and energy consumption. The innovation performance of high-polluting firms can be driven by different factors, such as market factor, competitor factor, government factor and so on [11]. Actually, high-polluting firms’ technological innovation may be affected by their proactive motivation or passive pressure. In terms of firms’ proactive motivation, corporate social responsibility requires firms to take responsibility for shareholders and environment during creating profits and bearing legal responsibilities [3]. In the signal theory, corporate social responsibility can convey information about sustainable strategies to all stakeholders, and represent the firms’ actual activities for environmental protection and social contribution [8]. Compared with other industries, corporate social responsibility performance of high-polluting firms can contain more information about corporate practices in environmental protection [12]. Therefore, high-polluting firms are willing to use technological innovation to support corporate practices in social contribution, and obtain more environmental benefits [13]. In terms of firms’ passive pressure, more and more people have paid attention to high-polluting industries due to pollutant emissions. Public attention, as the legitimacy-granting institution, can supervise the operations of high-polluting firms in real time [14]. Some behaviors of high-polluting firms will be restricted by public attention, such as energy consumption and pollutant emission. This passive pressure can promote high-polluting firms to make more contributions to environmental protection and social stability [15]. Based on the legitimacy theory, public attention can encourage high-polluting firms to concentrate on the practices of environmental protection [14]. Combining the proactive motivation and passive pressure, the association among corporate social responsibility, public attention and innovation performance has become a key factor in promoting the innovation performance of high-polluting firms, as well as resolving the conflict between environmental protection and environmental damage.

This paper explores the influence factors of innovation performance for high-polluting firms, and provides a better understanding of the relationship among corporate social responsibility, public attention and innovation performance. A panel data model is constructed based on a sample of Chinese listed firms in high-polluting industries from 2011 to 2016. The empirical results show that there is a positive association between corporate social responsibility and innovation performance, as well as public attention. Considering different ownership structure, the public attention of state-owned enterprises has a stronger impact on their innovation performance, indicating that passive pressure can bring more motivations of innovation performance to such high-polluting firms. Moreover, corporate social responsibility plays a stronger role in promoting the innovation performance of non-state-owned enterprises, and these firms actively convey information about corporate practices for environmental protection and social contribution. The differences between state-owned enterprises and non-state-owned enterprises demonstrate that the ownership structure of high-polluting firms may affect their technological innovations. State-owned enterprises with more resources and policy supports face more pressure from public attention. Non-state-owned enterprises relying on the external investments emphasize the importance of corporate social responsibility in reflecting their sustainable strategies. After introducing the pressure of regional economies, the innovation performance of high-polluting firms is hindered, but the impacts of corporate social responsibility and public attention are significantly strengthened, indicating that local governments are potential factors for innovation performance in high-polluting industries.

Our paper makes several contributions. First of all, it constructs a novelty research framework to explore the influence factors of innovation performance for high-polluting firms. We use corporate social responsibility and public attention to reveal the proactive motivation and passive pressure of high-polluting firms. Secondly, it explores the differences between state-owned enterprises and non-state-owned enterprises, such as the impacts of corporate social responsibility and public attention. High-polluting firms with different ownership structure can develop various innovation strategies based on their specific advantages, which will help them to get more environmental benefits. Finally, considering the role of local governments in China, the impact of regional economic pressure on innovation performance is analyzed at the province level, and it will help high-polluting firms cope with the conflict between macro-economic objectives and micro-environmental contributions.

The structure of this paper is as follows: Section 2 provides the background on innovation performance and the research hypotheses. Section 3 introduces the research methods, including samples, variables, and research models. Section 4 presents the empirical results, including the descriptive statistical analysis and the regression analysis. Section 5 provides the discussion of empirical results. The conclusions and recommendations are drawn in Section 6.

## 2. Background and Hypotheses

### 2.1. Innovation Performance

In existing studies, innovation performance can be driven by different factors, such as corporate characteristics, market demand, resources, partnerships and so on [11]. In terms of corporate characteristics, profitability is an important factor of financial performance for maintaining long-term development of enterprises, and it provides sufficient support for technological innovation. Rubera and Kirca pointed out that there is an association between financial performance and innovation performance, encouraging companies to make continuous attempts to technological innovation [16]. Huang and Li discussed the influence factors of innovation performance from the perspective of green innovation, proposing that corporate characteristics and social reciprocity can promote innovation performance [17]. Shan et al. found that entrepreneurial orientation can influence corporate innovation performance, and there is a significant inverted U-type relationship between entrepreneurial orientation and innovation performance [18]. In terms of market demand, Gök and Peker found market performance and multiple types of resources can promote innovation performance, generating more revenue for companies [19]. Gu et al. found that customer resource can promote innovation performance [20]. Among different resources, Ben Arfi et al. believed that external knowledge resources can influence the innovation performance of enterprises [21]. Serrano-Bedia et al. made an analysis of the relationship between knowledge resources and innovation performance from multiple dimensions, pointing out that there is a complementarity between internal and external knowledge resources on innovation performance [22]. In addition, partnerships of companies will influence innovation performance. Seo et al. found that the partnerships of R&D can influence innovation performance, and this relationship is different among companies with different technical levels [23]. Yu and Lee found that research organizations can bring more motivations to the innovation performance of partner enterprises [24]. Najafi-Tavani et al. analyzed the impact of collaborative innovation networks on innovation performance, proposing that partnerships between firms and research organizations can promote innovation performance under competitive environment [25].

Based on different forces of innovation performance, few studies have considered the impact of corporate social responsibility and public attention on innovation performance from the perspectives of proactive motivation and passive pressure, which also motivates our study.

### 2.2. Corporate Social Responsibility and Innovation Performance

In signal theory, corporate social responsibility can actively convey information about environmental protection and social contribution [26]. Owing to the negative impacts of high-polluting firms on ecological environment, such firms need to rely on technological innovations to improve the efficiency of resource utilization, in order to implement more environmental protection practices. Costa et al. analyzed the relationship between corporate social responsibility and innovation performance, pointing out that corporate social responsibility can promote innovation performance [27]. Cegarra-Navarro et al. found that the innovation performance can influence the practices of corporate social responsibility, and enterprises relying on innovation achievements will gain more public satisfaction [28]. Ratajczak and Szutowski discussed the associations between corporate social responsibility and innovation performance, pointing out that corporate social responsibility can directly influence innovation performance [29]. Marin et al. found that corporate social responsibility can significantly influence market competition, and innovation performance has become a moderator in competitions among different firms [30]. Martinez-Conesa et al. analyzed the relationship between corporate social responsibility and innovation performance, and found that corporate social responsibility can promote the growth of innovation capital [31]. Wu et al. found that corporate social responsibility can positively promote innovation performance with the regulating effect of public visibility [32]. Anser et al. analyzed the direct and mediating effect of firms’ innovation on corporate social responsibility, pointing out that innovation performance will directly influence the implementation of corporate social responsibility practices [33]. Ruggiero and Cupertino found that corporate social responsibility and financial performance can promote innovation performance, and corporate social responsibility can help firms cope with market competition [34]. Briones Peñalver et al. pointed out that the cooperative relationship among different firms can refine the relationship between corporate social responsibility and innovation performance, and corporate social responsibility will bring more motivations to firms’ innovation [35].

The environmental and social contributions of high-polluting firms need to be demonstrated through information disclosures. Corporate social responsibility, as an active signal, can demonstrate the motivation of high-polluting firms in improving resource utilization and reducing energy consumption. Therefore, we propose the following hypothesis:

**Hypothesis** **1.** *A firm’s innovation performance is positively related to corporate social responsibility in high-polluting industries.*

### 2.3. Public Attention and Innovation Performance

In the legitimacy theory, high-polluting firms are limited by social contract, and perform different socially expected activities for long-term development [14]. For high-polluting firms, public attention can directly reflect the social attention to environmental pollution, and become a kind of legitimacy-granting institutions. It is worth noting that there are few studies exploring the relationship between public attention and innovation performance, but the supervisory role of public attention has attracted the attention of many researchers. Webster believed that public attention owns a dual character in media environment, and provides different impacts among various social entities [36]. Simmons analyzed the relationship between public attention and accounting fraud, pointing out that increasing the degree of public attention could aggravate the punishment of accounting violations [37]. Bajo et al. discussed the impact of investors’ concerns about the process of IPO and proposed that public attention will encourage companies to display their specific characteristics [38]. With the development of environmental protection around the world, public attention has gradually become an important way to alleviate the problems of environmental pollution. Russell et al. found that public attention can influence policy issues, and create the dynamic process of policy-making [39]. Qin and Peng made an analysis of the impact of public attention on environmental topics and found that public attention will contribute to governments’ environmental management [40]. According to the legitimacy theory, public attention could pressure listed companies to invest more in environmental protection. Yu et al. analyzed the relationship between public attention and environmental performance of listed companies from the perspective of stakeholders, and believed that innovation strategies could mediate the impact of public attention on environmental performance [41]. Bird et al. pointed out that public attention can positively influence green innovation, promoting technological innovations to achieve higher practical value in environmental protection [42]. Marciano et al. analyzed the relationship between public attention and social economy, finding that public attention can promote product innovation [43]. Huang et al. (2016) explored the relationship between public attention and green innovation performance, and found that public attention can promote green innovation in manufacturing industries [44].

Based on the existing researches and the legitimacy theory, we infer that high-polluting firms suffer greater social pressure, and they need to improve the efficiency of resource utilization through technological innovation to satisfy social expectations. Therefore, we propose the following hypothesis:

**Hypothesis** **2.** *A firm’s innovation performance is positively related to public attention in high-polluting industries.*

### 2.4. Ownership, Corporate Social Responsibility and Public Attention

Besides corporate social responsibility and public attention, ownership characteristics of listed companies will also have an impact on the efficiency of resource acquisition and energy utilization. State-owned enterprises could obtain more government support and achieve sustainable development by financial resources and policy advantages [45]. Bi et al. discussed the influence factors of innovation performance and found that government factors can influence firms’ innovation, and bring different impacts on the innovation performance of state-owned and non-state-owned enterprises [46]. Jugend et al. found that government support can promote innovation performance more efficiently, and the ownership of firms can influence their innovation performance [47]. Guan and Pang analyzed the impact of industry characteristics on innovation performance in the Chinese market, pointing out that government subsidies will promote innovation performance of industrial enterprises [7]. Non-state-owned enterprises get more support from investors, and such firms are willing to convey information about corporate social responsibility in environmental protection. Long et al. analyzed the environmental performance of Korean companies in China, and found that non-state-owned enterprises pay more attention to environmental practices through technological innovation [48]. Xie et al. found that the ownership of technology-intensive firms could mediate the relationship between knowledge acquisition capabilities and innovation performance [49]. D’Agostino and Moreno discussed the relationship between inter-firm cooperation and innovation performance during different periods of economic crisis, and proposed that non-state enterprises rely on cooperation to promote their innovation performance [50]. The business environment of enterprises with different ownership structure is also an important factor in innovation performance, and non-state enterprises own more flexible innovation strategies than those of state-owned enterprises [11]. In addition, cooperative network can have a differential impact on the innovation performance of enterprises with different ownership structure [51].

State-owned enterprises often suffer the pressure from social expectations, and they demand technological innovation to satisfy the passive pressure. Non-state-enterprises are willing to convey information about environmental protection through technological innovation, and get more and more support from investors. Therefore, we propose the following hypotheses:

**Hypothesis** **3a.** *The positive relationship between public attention and innovation performance is stronger for the state-owned enterprises in high-polluting industries.*

**Hypothesis** **3b.** *The positive relationship between corporate social responsibility and innovation performance is stronger for the non-state-owned enterprises in high-polluting industries.*

**Figure 3:**
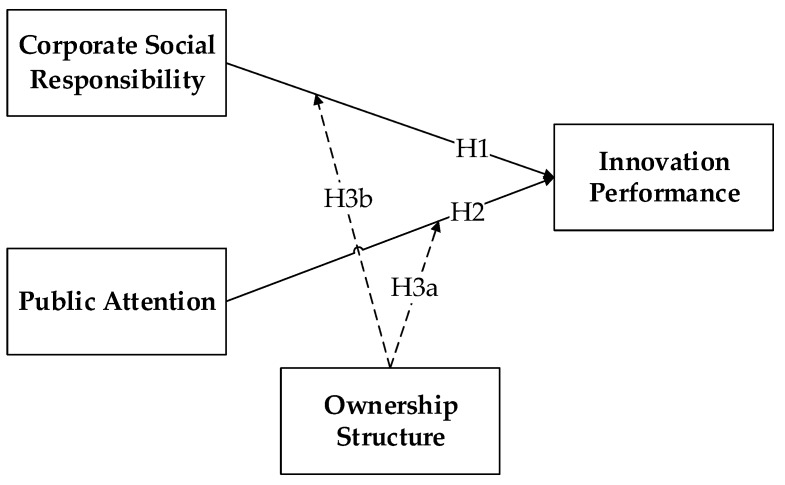
Research Model.

Few existing studies explore the influence factors of innovation performance from the perspective of proactive motivation and passive pressure. Based on the signal theory and the legitimacy theory, we explore the impact of corporate social responsibility and public attention on the innovation performance of high-polluting firms. The research model and hypotheses in this study are presented in Figure 3.

## 3. Research Methods

### 3.1. Samples and Data

Our paper aims to explore the impacts of corporate social responsibility and public attention on the innovation performance of high-polluting firms, in order to help such firms achieve long-term development. Combined with the characteristics of regional economy and industrial structure in China, high-polluting industries have become a factor of threatening the regional sustainable development. The innovation performance of high-polluting firms is relevant to many fields, including environmental protection, social relations and market competition. Compared with other industries, listed firms in high-polluting industries tend to receive higher level of attention from more people. Therefore, this paper chooses listed high-polluting firms as the research sample to analyse the influence factors of innovation performance.

Consistent with the “*Guidelines on environmental information disclosure of the listed companies*” published by the Ministry of Ecology and Environment of People’s Republic of China in 2010, we denote the following 16 industries as high-polluting industries, namely thermal power, steel, metallurgy, building materials, mining and so on. Empirically, we use the firm-level data of listed companies of Shanghai Stock Exchange and Shenzhen Stock Exchange, and use 20 two-digit industry codes of China Securities Regulatory Commission to obtain sample firms which belong to the above mentioned 16 kinds of high-polluting industries.

The data of corporate social responsibility come from Runling (RKS) CSR rating agency. The CSR scoring system of RKS evaluates CSR performance based on four dimensions: Macrocosm, Content, Technique and Industry. The CSR scoring data of RKS can be more objectively evaluated for corporate practices of Chinese listed companies in terms of corporate social responsibility, and the scoring indicators are more comprehensive [52]. The data of public attention come from Web Search Volume Index of Chinese Listed Companies (WSVI), which is an important database of China Research Data Services (CNRDS). This platform provides reliable support for many studies in the Chinese market. The regional economic data (regional GDP) are obtained from the website of the National Bureau of Statistics and Statistical Yearbooks of various provinces [4]. Date of innovation performance and corporate finance are derived from the China Stock Market and Accounting Research (CSMAR) database. CSMAR provides the financial data of Chinese listed companies. We are investigating a survival sample by deleting sample firms with incomplete data and also removing delisting companies. Finally, we obtained 738 firm-year observations of highly polluting industries in the Chinese market from 2011 to 2016.

### 3.2. Variables

#### 3.2.1. Dependent Variable

Innovation performance includes tangible state and intangible state. However, in high-polluting industries, where firms rely on physical products, innovations in intangible state could not adequately be related to their resource utilization. Referring to Rahko and Jiang et al. we use the number of patent applications to measure the innovation performance of high-polluting firms [53,54]. Considering periodicity of patent development, the average number of patent applications during the past three years is used as a proxy for innovation performance.

#### 3.2.2. Independent Variables

The independent variable, corporate social responsibility (CSR), represents the contribution of high-polluting firms to the environment, society and consumers. RKS CSR rating agency assesses the corporate social responsibility of Chinese listed companies, and compared with other institutions, the RKS scoring system better reveals the operations of these companies. Thus we select the overall scoring data of corporate social responsibility from RKS to measure the corporate social responsibility variable of high-polluting firms.

Public attention as the independent variable represents the importance that the whole society attaches to high-polluting firms. With the development of Internet technology, web search has become the main channel for obtaining information. The increasing online search volume of listed companies indicates that investors or potential investors have paid attention to these companies. The online search index of Chinese listed companies in the Web Search Volume Index of Chinese Listed Companies (WSVI) database is constructed based on the stock code, company abbreviation and company full name. In order to obtain public attention at the annual level, we use the median of online search index in the sample year to measure the public attention variable of high-polluting firms.

#### 3.2.3. Control Variables

Consistent with the previous literature of innovation performance, we employ control variables of organizational characteristics, financial information and corporate governance [33,45,54]. We select company age, company growth, leverage, return on assets, shareholding ratio of largest shareholder, proportion of independent directors as control variables. Company age (Age) measures the company’s maturity and is calculated as the natural logarithm of the duration from the initial public offering to the sample year. Company growth (Growth) measures the company’s growing potential, and we calculate it as the growth of operation revenue of listed companies. Leverage, the debt ratio of listed companies, measures financial distress and is calculated as the total debt over total assets. Return on assets (ROA), the profitability measure, is calculated as the net income divided total assets. Shareholding ratio of largest shareholder (First) is an important character of shareholding structure, and the proportion of independent directors among all directors (Independence) is a proxy for good corporate governance. Considering the characteristics of the Chinese market, we introduce the pressure of regional economies as a control variable at the macro-economic level, following Cheng and Liu (2018) [14]. The pressure of regional economies (GDP_Pressure) measures the regional economic growth pressure from local governments. Definitions of main variables are presented in Table 1.

### 3.3. Research Model

We use a panel data model to test the research hypotheses proposed in Section 2. The data of patent applications are countable, and its distribution is over-disperse. Considering the characteristics of innovation performance variable, the traditional OLS method may apply, while Poisson Regression and Negative Binomial Regression are two methods that are more appropriate. However, Poisson Regression requires the dependent variable to be equally distributed, and our sample data do not possess this distribution structure. Thus, Negative Binomial Regression is adopted for the dependent variable with over-dispersion [54]. We use Negative Binomial Regression to test the research hypotheses, and our specific regression model is as follows:

(1) $$\begin{array}{cc} Innovation_{i,t+1}= & \alpha_{0}+\beta_{1}CSR_{i,t}+\beta_{2}Attention_{i,t}+\beta_{3}Age_{i,t}+\beta_{4}Growth_{i,t}\\ + & +\beta_{5}Leverage_{i,t}+\beta_{6}ROA_{i,t}+\beta_{7}First_{i,t}+\beta_{8}Independence_{i,t}\\ + & Industry_{fixed\;effects}+Year_{fixed\;effects}+\varepsilon_{i,t} \end{array}$$

Equation (1) is the baseline model of this paper. Because it should take much time for getting the outcomes of innovation, the independent variables and control variables lag one year behind the dependent variable. $Innovation_{i,t+1}$ represents the innovation performance of firm *i* in year *t*+1. $CSR_{i,t}$ represents the corporate social responsibility of firm *i* in year *t*. $Attention_{i,t}$ represents the public attention of firm *i* in year *t*. $Age_{i,t}$ represents the company age of firm *i* in year *t*. $Growth_{i,t}$ represents the growth of operation revenue for firm *i* in year *t*. $Leverage_{i,t}$ represents the debt of firm *i* in year *t*. $ROA_{i,t}$ represents the profitability of firm *i* in year *t*. $First_{i,t}$ represents the shareholding structure of firm *i* in year *t*. $Independence_{i,t}$ represents the board structure of firm *i* in year *t*. $Industry_{fixed\;effects}$ and $Year_{fixed\;effects}$ are the industry fixed effects and the year fixed effects. $\varepsilon_{i,t}$ is the error term:

(2) $$\begin{array}{cc} Innovation_{i,t+1}= & \alpha_{0}+\beta_{1}CSR_{i,t}+\beta_{2}Attention_{i,t}+\beta_{3}Age_{i,t}+\beta_{4}Growth_{i,t}\\ + & \beta_{5}Leverage_{i,t}+\beta_{6}ROA_{i,t}+\beta_{7}First_{i,t}+\beta_{8}Independence_{i,t}\\ + & \beta_{9}GDP\_Pressure_{i,t}+Industry_{fixed\;effects}+Year_{fixed\;effects}+\varepsilon_{i,t} \end{array}$$

Innovation performance of listed companies may be influenced by regional economic development, and then we propose Equation (2) based on Equation (1). Equation (2) is to explore the influence of government factors on the innovation performance of high-polluting firms. Where $GDP\_Pressure_{i,t}$ represents the pressure of regional economies for firm *i* in year *t*.

## 4. Empirical Results

### 4.1. Descriptive Statistical Analysis

Table 2 provides results of descriptive statistics, including mean, median, standard deviation, minimum, and maximum value for each variable. Among all variables, the standard deviation of innovation performance variable (436.85) is the largest, and the interval between its maximum (5538) and minimum (0) is larger than other variables, indicating that this variable is over-dispersion. The standard deviation of corporate social responsibility variable (11.894) is relatively large, which indicates that the corporate social responsibility practices of high-polluting firms are significantly different. However, the distribution of public attention variable (0.609) is relatively concentrated, which indicates that the diversity of public attention for high-polluting firms is small. In the descriptive statistics of control variables, Growth variable and ROA variable are two variables worthy of discussion. The minimum values of Growth (−0.594) and ROA (−0.691) are negative, indicating that some high-polluting firms have suffered financial losses sometimes, and this state would also affect their sustainable strategies. In addition, the pressure of regional economies is a dummy variable, but its standard deviation (0.49) is higher than other control variables, which indicates that there is an imbalance of economic development in different cities of China.

Table 3 provides the results of Pearson correlation matrix and Variance Inflation Factor test. The correlation coefficient between CSR and Innovation is 0.274, indicating that high-polluting firms focusing on the environmental and social contributions often have more motivations for innovation performance.

The correlation coefficient between Attention and Innovation is 0.268, indicating that high-polluting firms with more social expectations have better innovation achievements. The absolute values of the correlation coefficient for all variables are lower than 0.5, so there is no multi-collinearity problem in the empirical model, ensuring that both the independent variables and control variables can effectively explain the dependent variable. In addition, the VIF values of all variables are less than 2, further indicating that there is no multi-collinearity problem in our panel data models, and this supports the reliability and accuracy of empirical results.

### 4.2. Regression Analysis

#### 4.2.1. Regression Analysis of Overall Sample

According to the panel data models constructed in Section 3, this paper first explores the impact of corporate social responsibility and public attention on innovation performance in the overall sample of China’s high-polluting firms. In the regression analysis, Negative Binomial Regression method is used to estimate the models constructed by Equation (1) and Equation (2). Industry and year fixed effects are controlled. The first part of regression analysis provides an overview of potential relationship among corporate social responsibility, public attention and innovation performance in the Chinese high-polluting industries.

Following the researches of [14,29], the innovation performance of high-polluting firms may be influenced by corporate social responsibility or public attention. We run regressions by adding the different kinds of variables step by step.

According to the regression results in Table 4, Model 1 explores the impact of corporate social responsibility and public attention on innovation performance without considering the control variables, and the coefficients of corporate social responsibility and public concern are both positive and statistically significant at 1%. This indicates that higher CSR scores are related to better innovation performance in high-polluting firms, and higher public attention is related to better innovation performance as well. Consistent with Bocquet et al., Model 2 explores the relationship between corporate social responsibility and innovation performance solely by adding firm characteristics as control variables [3]. The coefficient of corporate social responsibility is positive and statistically significant at 1%, indicating that there is a significant positive relationship between corporate social responsibility and innovation performance when considering the control variable. High-polluting firms concentrating on the environmental and social contributions prefer technological innovations for their long-term development. Model 3 explores the association between public attention and innovation performance solely with firm characteristics as control variables. The coefficient of public attention is positive and statistically significant at 1%, indicating that higher public attention is related to better innovation performance of high-polluting firms. Model 4 explores the relationship among corporate social responsibility, public attention, and innovation performance under Equation (1). The coefficients of corporate social responsibility and public attention are both positive and statistically significant at 1%. Comparing with the results of Model 1, the positive effects of corporate social responsibility and public attention are weakened, but they can still positively affect innovation performance, indicating that innovation performance will be motivated by the proactive motivation and passive pressure of high-polluting firms at the same time. Model 5 explores the impact of regional economies on the relationship among corporate social responsibility, public attention and innovation performance based on Equation (2). The coefficients of corporate social responsibility and public attention are both positive and statistically significant at 1%, while innovation performance is negatively related to the pressure of regional economies, which is consistent with the results of Cheng and Liu [14]. Comparing the results of Model 4 and Model 5, the positive effects of corporate social responsibility and public attention are strengthened, indicating that government factors could motivate high-polluting firms to concentrate on corporate social responsibility and public attention, so as to improve the utilization of resources through technological innovations. The results shown in Table 4 support our Hypothesis 1 and Hypothesis 2.

#### 4.2.2. Regression Analysis in SOE/non-SOE Subsamples

Some researchers suggest that high-polluting firms need to continuously acquire external resources for supporting technological innovation [55,56,57,58,59]. It is worth noting that state-owned enterprises in the Chinese market can obtain more policy support than non-state-owned enterprises, and their innovation strategies are usually determined by their ownership structure. To test our hypotheses 3a and 3b, we divide the overall sample into two sub-samples, namely the subsamples of state-owned enterprises and non-state-owned enterprises [60,61,62,63].

According to the regression results in Table 5, Models 6 and 7 explore the impact of corporate social responsibility and public attention on the innovation performance of state-owned enterprises respectively. The coefficients of corporate social responsibility and public concern are both positive and statistically significant at 1%. The state-owned enterprises with higher CSR scores or public attention prefer to invest more resources into technological innovation, and this is similar to the findings of Heli Wang et al. [64]. By comparing the results of Model 6 and Model 2, it can be found that the positive effect of corporate social responsibility of state-owned enterprises is weaker than that of total sample. However, the positive effect of public attention of state-owned enterprises is stronger than that of overall sample in the results of Model 7 and Model 3, which are similar to the results of Cheng and Liu [14].

As shown in Model 8, the coefficients of corporate social responsibility and public attention are both positive and statistically significant at 1%, which are consistent with the projections that corporate social responsibility and public attention can promote the innovation performance of state-owned enterprises. Comparing the results of Model 8 and Model 4, in the subsamples of state-owned enterprises, the effect of corporate social responsibility is weakened, but the effect of public attention is strengthened. This indicates that the passive pressure generated by public attention will provide more motivations for state-owned enterprises in technological innovation, consistent with Guan and Pang [7].

Model 9 explores the impact of regional economic development on the relationship among corporate social responsibility, public attention and innovation performance of state-owned enterprises. The coefficients of corporate social responsibility and public attention are both positive and statistically significant at 1%, while the innovation performance of these firms are negatively related to the pressure of regional economies. From the results of Model 9 and Model 8, the pressure of regional economies can enhance the impact of corporate social responsibility and public attention on the innovation performance of state-owned enterprises.

As the literature suggests that non-state-owned enterprises need to seek more investments from some potential investors, and their proactive motivation can change their technological innovation strategies [45,65].

In Table 6, Model 10 and Model 11 explore the impact of corporate social responsibility and public attention on the innovation performance of non-state-owned enterprises respectively. The coefficients of corporate social responsibility and public attention are both positive and statistically significant at 1%. The non-state-owned enterprises concentrating on corporate social responsibility or public attention will have more motivations for promoting technological innovation. As shown in Model 12, the coefficients of corporate social responsibility and public concern are both positive and statistically significant at 1%, indicating that corporate social responsibility and public attention can both drive the innovation performance of non-state-owned enterprises. From the results of Model 12 and Model 8, it is worth noting that the impact of CSR on the innovation performance of non-state-owned enterprises is significantly stronger than that of state-owned enterprises. However, the impact of public attention on the innovation performance of non-state-owned enterprises is significantly weaker than that of state-owned enterprises.

Model 13 explores the impact of regional economies on the relationship among corporate social responsibility, public attention and innovation performance of non-state-owned enterprises. The coefficients of corporate social responsibility and public concern are both positive and statistically significant at 1%, but there is no significant positive relationship between economic pressure and innovation performance. With the comparison between the results of Model 13 and Model 12, the pressure of regional economies cannot directly influence the innovation performance of non-state-owned enterprises.

These results proposed in Table 5 and Table 6 provide support for Hypothesis 3a and Hypothesis 3b, and high-polluting firms with different ownership structure should design various innovation strategies based on their specific advantages.

## 5. Discussion

Faced with the topics related to environmental deterioration, high-polluting firms need to take advantage of technological innovations to implement their sustainable strategies. In terms of social contributions, the corporate social responsibility of high-polluting firms can promote their innovation performance. Considering the social expectations of high-polluting industries, the public attention of high-polluting firms can motivate their innovation performance as well. Our empirical results are consistent with the findings of Cheng and Liu and Wu et al. [14,32]. Just like the discussion of government factors in Chen et al., the pressure of regional economies can inhibit the innovation performance of high-polluting firms, due to the pressure from local governments [45]. It is worth noting that this kind of pressure can strengthen the positive impacts of corporate social responsibility and public attention, so that high-polluting firms should deal with the relationship between productivity losses and environmental benefits.

Considering the impact of ownership characteristics, corporate social responsibility and public attention can promote the innovation performance of state-owned enterprises, which supports the findings of Han et al. [52]. Furthermore, the pressure of regional economies will inhibit the innovation performance of state-owned enterprises. This may be because local governments provide sufficient support for state-owned enterprises, and require them to achieve some economic goals. Faced with the pressure from local governments, state-owned enterprises may choose to expand the scale of production instead of promoting technological innovations. On the other hand, corporate social responsibility and public attention can also promote the innovation performance of non-state-owned enterprises. Comparing with the results of Huang et al., the pressure of regional economies cannot directly influence the innovation performance of non-state-owned enterprises [15]. In this scenario, non-state-owned enterprises have significant differences with state-owned enterprises in terms of resource acquisition and policy support. Non-state-owned enterprises prefer to convey information about sustainable strategies, and promote their innovation performance to obtain more investments from all stakeholders [65].

For high-polluting firms, technological innovation can help to implement sustainable strategies, and achieve long-term development. During the operations of high-polluting firms, their energy consumption and pollutant emission are closely related to environmental issues, and resolving these environmental problems will be the key step in their long-term development. High-polluting firms have to deal with the pressure of local governments, and satisfy the expectations of society. They need to rely on technological innovation to support their behaviors for environmental protection. During the operations of high-polluting firms, corporate social responsibility, as a proactive motivation, can convey information about environmental protection through technological innovation. Public attention, as a passive pressure, will bring more motivations for high-polluting firms to promote their innovation performance. From the perspective of sustainable development, it is necessary to take advantages of the relationship among corporate social responsibility, public attention and innovation performance for high-polluting industries. This relationship will provide guarantees for environmental benefits to all stakeholders of high-polluting firms.

## 6. Conclusions and Recommendations

### 6.1. Conclusions

Technological innovation is a key element of sustainable development for different firms, and has an important impact on resource utilization. High-polluting industries, as the main driving force of economic growth, have been closely linked to environmental issues for a long time. From the perspective of sustainable development, the debate between environment and economy is that whether we should protect the environment by reducing production, or destroy the environment for increasing revenue. In this situation, high-polluting firms need to explore the balance between profitability and sustainability, and the resource utilization and energy consumption become the key factors in this relationship. High-polluting firms should ameliorate their operations through technological innovation, thus achieving long-term development. Furthermore, the technological innovations of high-polluting firms can provide more environmental benefits and make more social contributions. The corporate social responsibility of high-polluting firms can convey information about corporate practices on environmental protection, and can be seen as a kind of signal for investors. High-polluting firms need to satisfy different social expectations, so that public attention has become a kind of legitimacy-granting institutions with informal supervision mechanism. Based on the signal theory and legitimacy theory, exploring the potential relationship among corporate social responsibility, public attention and innovation performance have become the determining factor of technological transition in the Chinese high-polluting industries.

In this study, we use a sample of listed high-polluting firms in the Chinese market from 2011 to 2016, and explore the impacts of corporate social responsibility and public attention on innovation performance. The Negative Binomial Regression method was chosen to empirically analyze the overall sample and subsamples (the subsample of state-owned enterprises and non-state-owned enterprises), and the impact of regional economies is also discussed. Empirically, we find that there is a positive relationship between corporate social responsibility and innovation performance of high-polluting firms, as well as public attention, indicating that the proactive motivation and passive pressure of high-polluting firms can promote their technological innovations. Corporate social responsibility is an important channel of delivering information about corporate activities for high-polluting firms, and reveals their social contribution and environmental protection. In order to gain more investors’ support, high-polluting firms need to try some new technological innovations to improve resource utilization, and reduce energy consumption. In addition, more and more people pay attention to the relationship between high-polluting industries and environmental issues. Public attention, as a passive pressure, has become a legitimacy-granting institution. Faced with this passive pressure, high-polluting firms should implement some sustainable strategies for reducing pollutant emission, thereby promoting the image of the central government. Furthermore, the pressure of regional economies cannot be ignored in the Chinese market, and the conflict between economy and environment may also have a corresponding impact on the innovation performance of high-polluting firms. Considering government factors, high-polluting firms should pay more attention to sustainable strategies, so as to promote technological innovations and maintain environmental benefits.

For high-polluting firms with different ownership structure, state-owned enterprises have obvious advantages in terms of the assets size and resource acquisition, so that they can receive sufficient support from local governments. Public attention will have a significant impact on the impression management of state-owned enterprises. Therefore, state-owned enterprises in high-polluting industries bear this passive pressure from social expectations. However, non-state-owned enterprises have difficulty in obtaining government resources, and their operations rely on the external investments. In order to gain investor’s support, non-state-owned enterprises need to convey information about social and environmental benefits to all stakeholders. In this situation, non-state-owned enterprises should pay more attention to corporate practices in environmental protection and social stability. In addition, state-owned enterprises suffer from the pressure of regional economies, which has a negative impact on their innovation performance. The pressure of regional economies can change the impacts of corporate social responsibility and public attention on the innovation performance of non-state-owned enterprises. High-polluting firms with different ownership structure should take advantage of proactive motivation or passive pressure to promote their innovation performance based on their specific advantages.

During exploring the influence factors of innovation performance in high-polluting industries, our results demonstrate that corporate social responsibility and public attention can significantly promote technological innovations. Corporate social responsibility can deliver more information about sustainable strategies of high-polluting firms, which is a signal of corporate environmental practices. Public attention, as a legitimacy-granting institution, can regulate the behaviors of high-polluting firms. The proactive motivation or passive pressure of high-polluting firms can promote technological innovation, and help to achieve the goal of improving resource utilization and reducing energy consumption.

### 6.2. Recommendations and Limitations

We focus on the innovation performance of high-polluting firms in our empirical analysis, and discuss the differences between the results of state-owned enterprises and non-state-owned enterprises. Considering the potential impact of local governments, high-polluting firms should deal with the relationship between environment and economy. Based on empirical results and conclusions, we propose the following recommendations:

First, high-polluting firms need to consider the influence factors of technological innovation from the perspective of firms’ proactive motivation. Corporate social responsibility can reflect the social contributions and environmental benefits of high-polluting firms for all stakeholders. High-polluting firms should balance the relationship between economic growth and environmental protection. Conveying information about innovation performance can help to get more and more support from investors, thus increasing the profitability of high-polluting firms.

Second, public attention can regulate the behaviors of high-polluting firms, and then motivate them to perform more environmental practices. High-polluting firms need to perform different socially expected activities, and take advantage of these social contracts to promote technological innovation. Understanding this passive pressure from social expectations can help high-polluting firms to achieve sustainable development.

There are also some limitations existing in this paper. In terms of time dimension, we select the data of listed companies from 2011 to 2016, and the time span may not fully demonstrate the relationship among corporate social responsibility, public attention and innovation performance. We obtain two subsamples, namely state-owned enterprises and non-state-owned enterprises. However, some non-state-owned enterprises are as good as state-owned enterprises, which makes the method of dividing samples imperfect. Combined with these limitations, future research on the innovation performance of high-polluting firms can consider a longer time span, and this will incorporate the impact of economic cycle into empirical analysis. In addition, the research sample can be divided into various subsamples by different methods, exhibiting the comprehensive relationship among corporate social responsibility, public attention and innovation performance of high-polluting firms.

## Figures and Tables

**Table 1:** Variable Definitions.

Variables	Type	Definition
Innovation	Dependent Variable	Innovation performance is measured by the average number of patent applications during the previous three years.
CSR	Independent Variables	CSR is calculated as CSR overall score from RKS rating agency.
Attention		Public attention is calculated as the natural logarithm of the median number of search volume index during the sample year plus one.
Age	Control Variables	Age is calculated as the natural logarithm of the duration from the initial public offering to the sample year.
Growth		Growth is calculated as the growth rate of operation revenue.
Leverage		Leverage is calculated as the total debt divided by the total assets.
ROA		ROA is the return on assets, which is calculated as the net income divided by the total assets.
First		First is calculated as the shareholding ratio of the largest shareholder.
Independence		Independence is the proportion of independent directors, which is calculated as the number of independent directors divided by the total number of directors on board.
GDP_Pressure		GDP_Pressure is a dummy variable which equals to 1 if the city-level GDP growth (where the firm was registered) is higher than the median of GDP growth in the locating province.

Note: CSR denotes the variable of corporate social responsibility; ROA denotes the variable of return on assets; GDP_Pressure denotes the pressure of regional gross domestic product.

**Table 2:** Descriptive Statistics.

Variables	Observations	Mean	Median	Standard Deviations	Min	Max
Innovation	738	90.330	22.333	436.850	0.000	5538.000
CSR	738	39.922	36.985	11.894	17.647	87.948
Attention	738	7.313	7.320	0.609	5.785	9.485
Age	738	2.570	2.639	0.331	1.099	3.178
Growth	738	0.122	0.060	0.402	−0.594	4.650
Leverage	738	0.485	0.487	0.198	0.034	1.112
ROA	738	0.040	0.034	0.068	−0.691	0.381
First	738	0.394	0.398	0.162	0.034	0.797
Independence	738	0.343	0.333	0.092	0.000	0.643
GDP_pressure	738	0.398	0.000	0.490	0.000	1.000

Note: CSR denotes the variable of corporate social responsibility; ROA denotes the variable of return on assets; GDP_Pressure denotes the pressure of regional gross domestic product.

**Table 3:** Pearson Correlation Matrix.

Variables	Innovation	CSR	Attention	Age	Growth	Leverage	ROA	First	Ind	GDP_P	VIF
Innovation	1.000										−
CSR	0.274 ***	1.000									1.20
Attention	0.268 ***	0.218 ***	1.000								1.16
Age	−0.006	0.201 ***	0.231 ***	1.000							1.18
Growth	−0.042	−0.063 *	−0.052	−0.103 ***	1.000						1.05
Leverage	0.032	0.074 **	−0.054	−0.070 *	0.049	1.000					1.36
ROA	−0.000	0.021	0.194 ***	−0.064 *	0.129 ***	−0.463 ***	1.000				1.42
First	0.257 ***	0.275 ***	−0.001	0.003	−0.013	0.022	0.038	1.000			1.11
Ind	−0.206 ***	−0.098 ***	0.008	−0.138 ***	0.016	−0.147 ***	0.068 *	−0.064 *	1.000		1.06
GDP_P	−0.085 **	−0.013	−0.110 ***	−0.183 ***	0.027	0.014	−0.075 **	−0.111 ***	0.109 ***	1.000	1.08

Note: ***, **, * represent the significance at the level of 1%, 5%, 10% respectively. CSR denotes the variable of corporate social responsibility; ROA denotes the variable of return on assets; Ind denotes the variable of Independence; GDP_Pressure denotes the pressure of regional gross domestic product. VIF is the abbreviation for variance inflation factor.

**Table 4:** The Regression Results of the Total Sample.

Variables	Total Sample				
	Model 1	Model 2	Model 3	Model 4	Model 5
CSR	0.0227 ***	0.0221 ***		0.0179 ***	0.0193 ***
	(6.11)	(5.94)		(5.01)	(5.28)
Attention	0.8362 ***		0.8861 ***	0.8350 ***	0.8439 ***
	(8.66)		(8.47)	(8.02)	(8.31)
Age		−0.0731	−0.2214	−0.3146 **	−0.4064 ***
		(−0.50)	(−1.50)	(−2.14)	(−2.66)
Growth		0.0589	0.1267	0.1176	0.1221
		(0.77)	(1.25)	(1.20)	(1.28)
Leverage		1.1419 ***	0.7427 **	0.6439 **	0.5803 **
		(3.87)	(2.50)	(2.18)	(2.01)
ROA		6.2289 ***	3.0363 ***	3.1031 ***	2.8961 ***
		(5.95)	(2.73)	(2.85)	(2.79)
Fist		2.0981 ***	2.8632 ***	2.5301 ***	2.4575 ***
		(6.92)	(9.30)	(8.24)	(7.96)
Independence		0.1448	−0.1272	0.1324	0.3080
		(0.28)	(−0.27)	(0.28)	(0.65)
GDP_Pressure					−0.2725 ***
					(−3.18)
Constant	−4.1837 ***	−0.0573	−4.9195 ***	−5.0965 ***	−4.8771 ***
	(−5.75)	(−0.12)	(−5.56)	(−5.88)	(−5.78)
Industry-fixed effects	Yes	Yes	Yes	Yes	Yes
Year-fixed effects	Yes	Yes	Yes	Yes	Yes
Observations	615	615	615	615	615
Wald $\chi^{2}$	1544.34	1670.16	2390.24	2751.17	2453.09
Pseudo R^2^	0.1077	0.1103	0.1184	0.1217	0.1231

Note: figures in parentheses are the z statistics of estimated coefficients; ***, **, * represent the significance at the level of 1%, 5%, 10% respectively. CSR denotes the variable of corporate social responsibility; ROA denotes the variable of return on assets; GDP_Pressure denotes the pressure of regional gross domestic product.

**Table 5:** The Regression Results of State-Owned Enterprises.

Variables	Subsample of State-Owned Enterprises			
	Model 6	Model 7	Model 8	Model 9
CSR	0.0185 ***		0.0132 ***	0.0147 ***
	(4.05)		(3.08)	(3.42)
Attention		0.8890 ***	0.8453 ***	0.9158 ***
		(7.27)	(6.86)	(7.63)
Age	0.2624	0.2117	0.1535	0.0705
	(1.45)	(1.15)	(0.85)	(0.38)
Growth	0.0826	0.1269	0.1331	0.1385
	(1.07)	(1.18)	(1.23)	(1.31)
Leverage	1.2246 ***	0.7017 *	0.5822	0.5600
	(3.43)	(1.95)	(1.60)	(1.59)
ROA	6.7477 ***	3.3723 ***	3.3687 ***	2.9478 **
	(6.12)	(2.66)	(2.71)	(2.49)
Fist	1.6054 ***	2.6256 ***	2.3483 ***	2.4761 ***
	(3.55)	(5.85)	(5.11)	(5.53)
Independence	0.6102	0.1190	0.1691	0.4142
	(0.95)	(0.20)	(0.28)	(0.68)
GDP_Pressure				−0.4260 ***
				(−4.32)
Constant	−0.7999	−6.0469 ***	−6.0653 ***	−6.3880 ***
	(−1.51)	(−5.62)	(−5.69)	(−6.24)
Industry-fixed effects	Yes	Yes	Yes	Yes
Year-fixed effects	Yes	Yes	Yes	Yes
Observations	455	455	455	455
Wald $\chi^{2}$	1042.42	1454.91	1550.51	1706.69
Pseudo R^2^	0.1204	0.1306	0.1323	0.1356

Note: figures in parentheses are the z statistics of estimated coefficients; ***, **, * represent the significance at the level of 1%, 5%, 10% respectively. CSR denotes the variable of corporate social responsibility; ROA denotes the variable of return on assets; GDP_Pressure denotes the pressure of regional gross domestic product.

**Table 6:** The Regression Results of Non-State-Owned Enterprises.

Variables	Subsample of Non-State-Owned Enterprises			
	Model 10	Model 11	Model 12	Model 13
CSR	0.0267 ***		0.0260 ***	0.0254 ***
	(4.47)		(4.48)	(4.44)
Attention		0.4363 ***	0.3949 ***	0.4177 ***
		(2.94)	(2.88)	(3.01)
Age	−0.0720	−0.0881	−0.2817	−0.2346
	(−0.26)	(−0.29)	(−0.96)	(−0.76)
Growth	0.3403	0.4511 **	0.3233	0.3311
	(1.44)	(2.11)	(1.54)	(1.60)
Leverage	0.2533	0.0880	0.0025	0.0548
	(0.50)	(0.16)	(0.00)	(0.10)
ROA	2.7422 *	1.2130	1.3833	1.4109
	(1.68)	(0.73)	(0.86)	(0.86)
Fist	1.9059 ***	2.4799 ***	2.0020 ***	2.1129 ***
	(4.78)	(6.18)	(5.09)	(5.27)
Independence	−0.7375	−1.0642	−0.3574	−0.3772
	(−1.10)	(−1.50)	(−0.52)	(−0.55)
GDP_Pressure				0.0937
				(0.71)
Constant	0.3725	−1.7365	−2.1909 *	−2.5511 *
	(0.35)	(−1.26)	(−1.77)	(−1.95)
Industry-fixed effects	Yes	Yes	Yes	Yes
Year-fixed effects	Yes	Yes	Yes	Yes
Observations	170	170	170	170
Wald $\chi^{2}$	2925.01	2256.28	2693.59	2712.33
Pseudo R^2^	0.1468	0.1381	0.1508	0.1511

Note: figures in parentheses are the z statistics of estimated coefficients; ***, **, * represent the significance at the level of 1%, 5%, 10% respectively. CSR denotes the variable of corporate social responsibility; ROA denotes the variable of return on assets; GDP_Pressure denotes the pressure of regional gross domestic product.
